# Notes and News

**Published:** 1898-05-14

**Authors:** 


### Notes and News.

(Collected and Communicated.)

HOSPITAL MEETINGS.

Alexandra Hospital for Children.—Annual Meeting, Thursday, May 19th, 4.30 p.m., at the Hospital.

Brompton Hospital for Consumption.—Annual Meeting, Thursday, May 26th, 4 p.m., at the Hospital.

Victoria Hospital for Children.—Annual Meeting, Wednesday, May 18th, 5 o'clock.

Liverpool is determined not to rest satisfied till it has provided 700 beds for the accommodation of infectious cases arising in the city.

The Prince of Wales, in his capacity of vice-patron of the University College Hospital, has promised to lay the foundation-stone of the new building on June 21st.

The Duchess of Newcastle will give a concert at 11, Hill Street, on June 28th, in aid of the Victoria House for Invalid Children at Margate.

The inordinate amount of illness among the naval cadets of the *Britannia* is causing pertinent questions in the House as to whether improper feeding has anything to do with the production of so much sickness.

The Victoria Wing of the Morley Convalescent Home has just been formally opened by the Lord Chancellor. The Prince of Wales telegraphed expressing his great interest in the institution.

Owing to the fact that the London Hospital provides several Jewish wards, the Jewish community in London should liberally respond to the special appeal on behalf of this hospital.

A monthly journal dealing with the diseases of warm climates will shortly be started in London under the editorship of Mr. James Cantlie and Dr. W. J. Simpson. Many influential men interested in tropical work have promised their support, and we should anticipate for the new venture a career of much usefulness. It is said that there are nearly 6,000 medical men holding British diplomas practising their profession outside the British Isles, most of whom reside in warm climates, so that the new paper ought not to be wanting in a *clientèle*. The *Journal of Tropical Medicine* will be published by Messrs. Bale, Sons, and Danielsson.

The Vaccination Bill was read a second time on Monday last.

A bronze bust of the late James Greig-Smith, who was for many years surgeon to the Bristol Royal Infirmary, has been placed in the Bristol Museum and Library.

The annual festival dinner in aid of the Royal Hospital for Incurables is fixed to take place on Tuesday, 7th June, at the Hotel Metropole, the chair being taken by Mr. Alderman George Wyatt Truscott.

A concert is to take place on the 26th inst. at Stafford House in aid of the Princess May's Sailors' Reading-rooms and Home. Madame Belle Cole, Madame Mary Davies, Mr. Ben Davies, and many other well-known artistes have kindly promised their services.

A musical afternoon and sale of work is to be held on May 17th at the Passmore Edwards Settlement, Tavistock Place, at half-past two, in aid of the funds of the Ladies' Samaritan Society of the National Hospital for the Paralysed and Epileptic.

A "Bereaved Mother" has complained in the *Morning Post* of that kind of appeal on behalf of hospitals which is based on the birth of babies, as published in the papers. She states that in an appeal to her on behalf of a children's cot in a hospital the exact date of her baby's birth is given. The baby having since died, she says she considers the appeal is couched in terms calculated to open old wounds in an unnecessary manner.

We are apt to associate the work of district visiting with church work and with the distribution of religious tracts. The new district visitor, however, is to be engaged in preaching that new religion which is peculiarly the product of this end of the present century, namely, the gospel of hygiene. She is to be a well-trained woman, with nursing experience, and grounded in the elements of hygiene, and her work will be to go from house to house and talk with the women on the care of their children, the necessity of cleanliness, the preparation of food, the virtues of fresh air, and all such matters as concern the hygiene of the house and of the person. "Health missioners" they are to be called. They exist in Manchester, and Dr. Allan, medical officer of health for the Strand, now advocates their introduction into his district.

The report of Dr. William Collingridge, medical officer for the Port of London, which has just been published, shows the active supervision which is being constantly exercised over the sanitary conditions under which trade is being carried on upon our great waterway. During the six months dealt with, 14,795 vessels were inspected. Certain difficulties appear to have been met with in regard to the sanitary condition of some of the barges or lighters trading on the river, for while these craft are not actual dwellings, inasmuch as no one is supposed to live or sleep on board, and are thus to a certain extent outside the Act, it seems none the less important that their sanitary condition should be under proper control. As a result of the medical inspection at Gravesend of vessels entering the port, 30 cases of infectious disease were discovered, the whole number of such cases dealt with by the authority during the half-year being 85.

